# Differential expression and tumorigenic function of neurotensin receptor 1 in neuroendocrine tumor cells

**DOI:** 10.18632/oncotarget.4745

**Published:** 2015-07-27

**Authors:** Ji Tae Kim, Jing Li, Jun Song, Eun Y. Lee, Heidi L. Weiss, Courtney M. Townsend, B. Mark Evers

**Affiliations:** ^1^ Markey Cancer Center, University of Kentucky, Lexington, KY, USA; ^2^ Department of Surgery, University of Kentucky, Lexington, KY, USA; ^3^ Department of Pathology and Laboratory Medicine, University of Kentucky, Lexington, KY, USA; ^4^ Department of Surgery, University of Texas Medical Branch, Galveston, TX, USA

**Keywords:** neuroendocrine tumor, neurotensin receptor 1, promoter methylation, cell growth, cell migration

## Abstract

Neurotensin (NTS), localized predominantly to the small bowel, stimulates the growth of a variety of cancers, including neuroendocrine tumors (NETs), mainly through its interaction with the high-affinity NTS receptor 1 (NTSR1). Here, we observed increased expression of NTSR1 in almost all tested clinical NET samples, but not in normal tissues. Through RT-PCR analysis, we found that the expression of NTSR1 and NTSR2 was either variable (NTSR1) or absent (NTSR2) in human NET cell lines. In contrast, NTSR3 and NTS were expressed in all NET cells. Treatment with 5-aza-2′-deoxycytidine, a demethylating agent, increased levels of NTSR1 and NTSR2 suggesting that DNA methylation contributes to NTSR1/2 expression patterns, which was confirmed by methylation analyses. In addition, we found that knockdown of NTSR1 decreased proliferation, expression levels of growth-related proteins, and anchorage-independent growth of BON human carcinoid cells. Moreover, stable silencing of NTSR1 suppressed BON cell growth, adhesion, migration and invasion. Our results show that high expression of NTSR1 is found in clinical NETs and that promoter methylation is an important mechanism controlling the differential expression of NTSR1 and silencing of NTSR2 in NET cells. Furthermore, knockdown of NTSR1 in BON cells suppressed oncogenic functions suggesting that NTSR1 contributes to NET tumorigenesis.

## INTRODUCTION

Neurotensin (NTS), a 13-amino acid peptide, functions as a primary neurotransmitter as well as a neuromodulator in the central nervous system (CNS) and as a hormone in the periphery [1–3]. NTS contributes to numerous physiologic functions in the gastrointestinal (GI) tract including GI secretion, gut motility, and growth of various normal tissues [1, 2]. Moreover, NTS stimulates the growth of several cancer types including neuroendocrine tumors (NETs) that, compared to other cancers, are increasing in incidence [1, 3, 4].

The actions of NTS are mediated through three receptors (i.e., NTSR1, NTSR2 and NTSR3/sortilin), named according to the order in which they were cloned [2, 3]. In particular, high-affinity NTSR1, which is found in various regions of the CNS, in the small and large intestine, and in a variety of solid tumors, is considered a predominant mediator of the effects of NTS on cell proliferation, migration, and invasion [3, 5]. In contrast, the low-affinity NTSR2, which shares 60% homology with NTSR1, demonstrates a more localized distribution; the expression of NTSR2 has been recently reported in prostate cancers and B cell lymphomas [6, 7]. Different from NTSR1 and NTSR2, which are G protein-coupled receptors, NTSR3/sortilin is a single transmembrane receptor, which binds various neurotrophic factors and neuropeptides and is not specific for NTS [3, 8].

Epigenetic alterations involving DNA methylation or histone modifications can vary the expression patterns of genes that are important for cancer development and progression [9–11]. For example, it is well known that hypermethylation of CpG islands in the promoter region of tumor suppressor genes results in gene silencing, which can lead to the facilitation of tumor progression in certain tissues [9, 12]. In addition, DNA hypomethylation, which is also observed in many cancers, induces transcriptional activation of oncogenes and contributes to cancer progression [9, 13, 14].

Diverse expression levels of the NTSRs, especially increased expression of NTSR1, have been reported in various types of cancers (e.g., colon, pancreas, breast, lung and prostate) [15–21]; however, the molecular mechanisms for this altered expression pattern are not entirely known. Although NTS can stimulate the growth of NET cells [4], the expression profiles and cellular functions of the NTSRs have not been well-delineated in NETs. In our present study, we analyzed the expression of NTSR1 protein in normal and NET tissues for GI, lung and thymus, and endogenous expression of NTSRs and transcriptional repression of NTSR1 and NTSR2 genes in NET cell lines. We demonstrate the epigenetic alteration of NTSR1 and NTSR2 by methylation analyses of their promoters in NET cells and in clinical tissues. Furthermore, we show that NTSR1 knockdown suppresses cell proliferation, anchorage-independent growth, attachment, migration and invasion of NET cells.

## RESULTS

### Expression of NTSR1 in normal and NET tissues by immunohistochemistry

**Figure 1:**
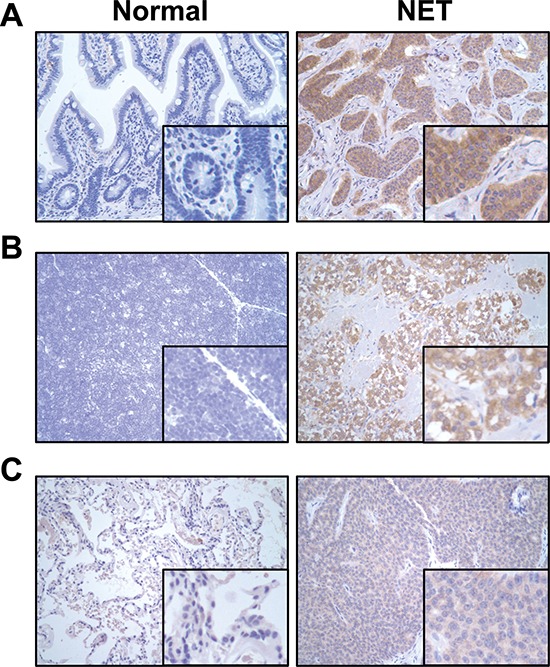
Expression of NTSR1 in normal and clinical NET tissues Immunohistochemical analysis of NTSR1 was performed in human normal and NET tissues. Representative images for NTSR1 protein expression in GI **A.** thymus **B.** and lung **C.** were shown at 200 × magnification. While the staining of NTSR1 was absent or barely detected in normal tissues (left), strong NTSR1 labeling was observed in clinical NET tissues (right).

NTS and NTSR1 complexes have been frequently observed in progression of several types of tumors. Although the expression of NTS which is mainly released by endocrine cells in the small bowel is broadly known in NETs, NTSR1 expression has not been well-studied in NETs. To evaluate the expression of NTSR1, immunohistochemical analysis was performed in clinical NET patient samples (i.e., 12 GI, 2 thymus and 6 lung NET tissues) used in our previous study [22]. Compared to normal tissues (5 GI, 2 thymus and 5 lung tissues) in which NTSR1 was not or barely detected, increased expression of NTSR1 was observed in all tested GI (12 of 12 NETs, Fig. 1A), and thymus (2 out of 2 NETs, Fig. 1B), and a majority of lung (5 out of 6 NETs, Fig. 1C) NET samples. These data suggest that NTSR1 is highly expressed in NETs but not in normal tissues.

### Endogenous expression of NTS and NTSRs, and induction of NTSR1 and NTSR2 by 5-aza-CdR treatment in NET cells

**Figure 2:**
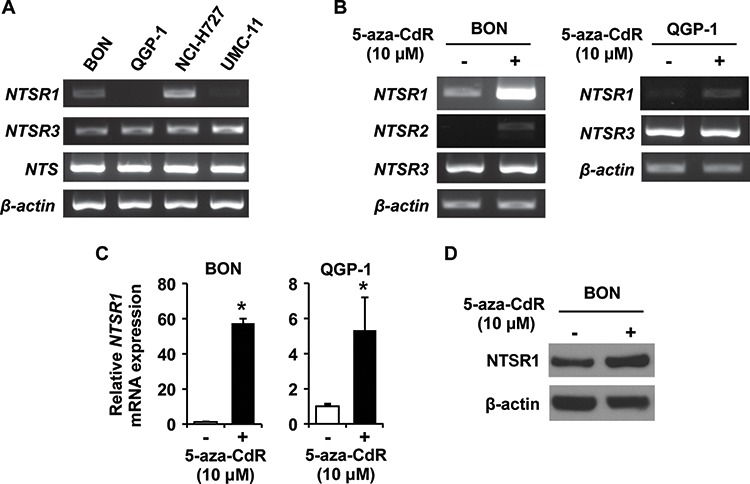
Expression analysis of NTSRs in endogenous and 5-aza-CdR treated NET cell lines **A.** RT-PCR analysis of *NTS*, *NTSR1, NTSR2, NTSR3* and *β-actin* expression in NET cells. **B.** RT-PCR analysis of *NTSRs* and *β-actin* expression in BON and QGP-1 cells treated with 0 (DMSO) or 10 μM 5-aza-CdR. The media containing 5-aza-CdR were replaced every 24 h for 4 d. **C.** Quantitative RT-PCR (qRT-PCR) analysis confirmed that treatment with 5-aza-CdR increased the expression of *NTSR1* gene in BON and QGP-1 cells. The reaction was performed using a TaqMan Gene Expression Master Mix and TaqMan probes for human NTSR1 and GAPDH as internal control (Applied Biosystems). Expression levels were assessed by evaluating threshold cycle (Ct) values. The relative amount of mRNA expression was calculated by the comparative ΔΔCt method (*p < 0.05 vs. DMSO). **D.** Western blot analysis showing induction of NTSR1 by 5-aza-CdR treatment for 96 h in BON cells. The protein extracts for cell lysates were analyzed with the indicated antibodies. β-actin was used as a loading control.

To elucidate expression profiles of NTS signaling components in NET cells, we first analyzed the expression of NTS and NTSRs in four human NET cell lines (BON, QGP-1, NCI-H727 and UMC-11) by RT-PCR. Expression of NTS and NTSR3 mRNA was noted in all four cell lines (Fig. 2A); in contrast, NTSR2 expression was not detected (data not shown). Variable expression of NTSR1 was noted with the greatest expression in NCI-H727 cells and moderate expression in BON; very little or no NTSR1 transcripts were demonstrated in QGP-1 and UMC-11 cells (Fig. 2A).

Previously, we found that repression of Wnt inhibitory genes (*SFRP-1*, *Axin-2*, *DKK-1*, *DKK-3* and *WIF-1*) results from promoter methylation or histone modification in NET cells [22]. To determine whether alterations in expression levels of NTSR1 and NTSR2 were due to epigenetic mechanisms (e.g., promoter methylation), we treated BON and QGP-1 cells with a demethylating agent, 5-aza-2′-deoxycytidine (5-aza-CdR), and examined the expression of NTSRs using RT-PCR (Fig. 2B). Treatment with 5-aza-CdR increased the expression of NTSR1 and NTSR2 in BON and the expression of NTSR1 in QGP-1 cells. To confirm these results, mRNA expression levels were also investigated by qRT-PCR (Fig. 2C). Treatment of the cells with 5-aza-CdR resulted in an approximate 57-fold induction of NTSR1 expression in BON and an approximate 5-fold induction in QGP-1 cells. Western blot analysis confirmed that the level of NTSR1 protein was augmented in BON cells treated with 5-aza-CdR (Fig. 2D). Collectively, our findings of mRNA and protein expression suggest that NTSR1 and NTSR2 are targets of epigenetic modulation through methylation in NET cells.

### Methylation status of NTSR1 and NTSR2 promoters in NETs

**Figure 3:**
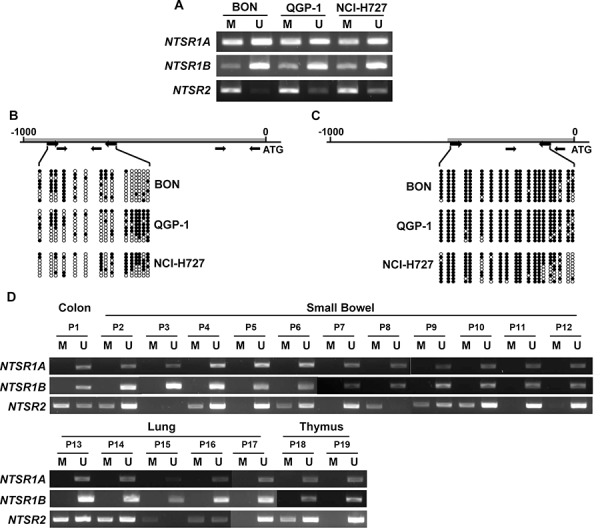
DNA methylation analysis of the *NTSR1* and *NTSR2* promoters in NETs **A.** MSP analysis of *NTSR1* and *NTSR2* promoters with respective two primer pairs (NTSR1A and NTSR1B) and primers (NTSR2) specific for the methylated (M) and unmethylated (U) DNA in three NET cell lines. The PCR products were visualized by 2% agarose gel. **B.** Bisulfite genomic sequencing analysis of *NTSR1* promoters in BON, QGP-1 and NCI-H727 cells. Each row of circles represents the DNA sequence of an individual clone; closed and open circles indicate methylated and unmethylated CpG sites, respectively. Bold grey lines are candidate CpG islands searched by the software of Applied Biosystems. The thicker and upper, and thinner and lower arrows below the CpG islands represent the primers for bisulfite sequencing and MSP, respectively. **C.** Bisulfite genomic sequencing analysis of *NTSR2* CpG islands in the NET cells. **D.** MSP analysis of *NTSR1* and *NTSR2* promoters with the same primers described above in clinical NET samples.

To test whether the induction of NTSR1 and NTSR2 by 5-aza-CdR was due to promoter methylation, we examined the methylation status of the 5′ regions of these genes using methylation-specific PCR (MSP) and bisulfite sequencing in three NET cell lines. Using two primer pairs, MSP analysis showed partial methylation of the NTSR1 promoter in all tested cell lines (Fig. 3A). The methylation profile of the 5′ region of NTSR1 was further analyzed by the direct sequencing of the MSP products (Supplementary Fig. 1A) and bisulfite sequencing (Fig. 3B). The CpG sites of the NTSR1 promoter were partially methylated in tested NET cells. Moreover, promoter methylation of NTSR2 was also noted by MSP analysis (Fig. 3A). Similar sequencing analyses for the NTSR2 promoter confirmed hypermethylation of the CpG islands consistent with the MSP data (Fig. 3C and Supplementary Fig. 1B).

CpG island methylation of NTSR1 and NTSR2 was further investigated in the above clinical specimens used in immunohistochemical analyses. By MSP analysis, methylation of the NTSR1 promoter was not noted in any of the NET specimens, and methylation of NTSR2 was observed in 12 out of 19 NET samples (Fig. 3D). Surprisingly, promoter methylation of NTSR1 was shown in 11 out of 12 normal tissues samples (Supplementary Fig. 2). These data demonstrate that reduction or silencing of NTSR gene expression was strongly associated with DNA methylation of the respective gene promoters in NET cell lines and patient samples. In particular, the absence of NTSR1 promoter methylation is in line with NTSR1 protein expression (Fig. 1) and leads to a strong expression of the protein in tested clinical NET samples. In addition, Dong *et al*. [25, 26] in our laboratory reported that DNA methylation contributes to NTS expression in human liver and colon cancer cells. Therefore, based on our current study and previous reports, it is clear that DNA methylation can control NTS signaling by regulation of expression levels for the agonist (i.e., NTS) and its receptors (NTSR1 and NTSR2).

### NTSR1 knockdown inhibits NET cell growth and migration

**Figure 4:**
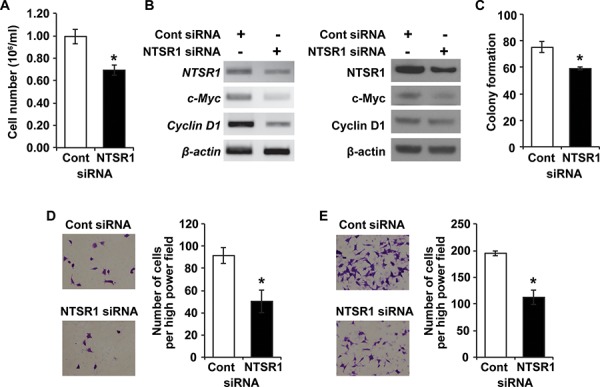
Knockdown of NTSR1 affects NET cell growth and migration **A.** Equal numbers of BON cells transfected with siRNA against non-targeting control or NTSR1 were plated in 24 well plates. The cell numbers were counted after 48 h incubation using a cell counter (*p < 0.05 vs. control siRNA). **B.** RT-PCR (left) and western blot (right) analyses showing expression of NTSR1, c-Myc and Cyclin D1 in BON cells transfected with control or NTSR1 siRNA. β-actin was used as a loading control. **C.** The number of colonies compared with the control siRNA in soft agar assay. Colony formation of representative control or NTSR1 knockdown BON cells was assessed over a period of 4 wks (*p < 0.05 vs. control siRNA). **D.** Boyden chamber migration assay with type I collagen-coated Transwells was carried out with control or NTSR1 knockdown BON cells over 24 h. Phase-contrast microscopic images (left) and quantification of migrated cells (right), which were counted in four different fields with an inverted microscope (*p < 0.05 vs. control siRNA), are shown. **E.** Transwell migration assay performed with respective siRNA-transfected BON cells over 48 h as described above.

Recently, we showed that expression and secretion of NTS are directly regulated by the Wnt/β-catenin pathway in NET cells [4]. We also found that inhibition of NTS signaling suppressed cell proliferation and anchorage-independent growth in these cells [4]. To further delineate the possible proliferative effect of NTSR1, we used small interfering RNA (siRNA) against NTSR1 in BON cells, which express NTSR1 mRNA and have been widely utilized as a novel carcinoid cell model [27]. Compared with cells transfected with non-targeting control, siRNA-mediated knockdown of NTSR1 suppressed cell proliferation (Fig. 4A). In addition, NTSR1 knockdown significantly inhibited the expression of c-Myc and Cyclin D1 which play integral roles in cell proliferation (Fig. 4B) [4, 28, 29]. Next, we performed soft agar assays to determine the effect of NTSR1 knockdown on anchorage-independent growth. The number of BON cell colonies transfected with NTSR1 siRNA was significantly lower than those of control cells (Fig. 4C). These findings suggest that a growth-stimulating function of NTS may be mediated mainly by NTSR1 in NET cells.

In addition to cell growth, activation of NTSR1 induces cell migration, invasion, and metastasis in head and neck squamous cell carcinomas, glioblastomas and breast cancer cells [19, 30, 31]. Based on these studies, we evaluated the migration of BON cells transfected with NTSR1 siRNA using a Boyden chamber migration assay with type I collagen-coated Transwells. Knockdown of NTSR1 decreased the migratory potential of BON cells at 24 (Fig. 4D) and 48 h (Fig. 4E), respectively. Additionally, the effect of pharmacologic blockade of NTSR1 using SR-48692 on NET cell migration was assessed by Transwell migration assays. Treatment with SR-48692, which represses NET cell growth [4], significantly decreased BON cell migration in a dose-dependent manner (Supplementary Fig. 3A). Taken together, these findings show that knockdown of NTSR1 through siRNA or treatment with a selective NTSR1 antagonist inhibits cell growth and migration of NET cells.

### Stable silencing of NTSR1 suppresses proliferation, adhesion, migration and invasion in NET cells

**Figure 5:**
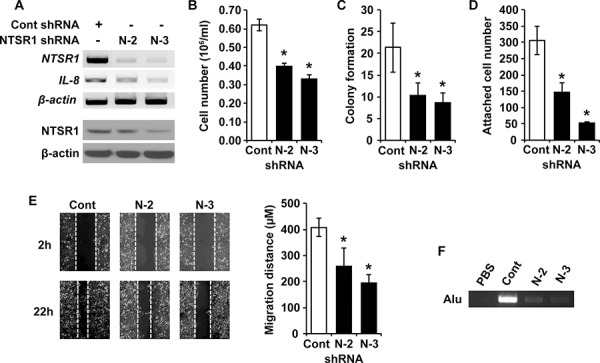
Stable silencing of NTSR1 suppresses cell growth, adhesion, migration and invasion of NET cells **A.** RT-PCR (upper) and western blot (bottom) analyses showing NTSR1 expression in stable non-targeting control or two NTSR1 knockdown BON cell clones (N-2 and N-3). **B.** The stable BON cell clones were incubated for 48 h; cell numbers were counted using a cell counter (*p < 0.05 vs. control shRNA). **C.** The number of colonies compared with control shRNA in soft agar assay. Colony formation of representative BON stable cell clones was assessed over a period of 4 wks (*p < 0.05 vs. control shRNA). **D.** The same number of stable BON cells was added onto type I collagen-coated plate for 15 min. The attached cells were fixed, and then stained with crystal violet. The number of attached cells was counted and the mean values were determined (*p < 0.05 vs. control shRNA). **E.** Phase-contrast microscopic images showing stable BON cell clones 2 h (upper) and 22 h (bottom) after removing the Culture-Insert (Ibidi, Munich, Germany). Wounding migration assay performed with control and the two BON cell clones expressing NTSR1 shRNA over 22 h. Quantification was carried out by measuring the migrated distance (right, *p < 0.05 vs. control shRNA). **F.** Reduction of invasive activity by stable silencing of NTSR1 in BON cells. To determine the invasive effect of NTSR1, human Alu sequence PCR-based assay was performed. Genomic DNA isolated from lower CAMs was used as a template of PCR amplification. PBS was used as negative control. The PCR product was electrophoresed on a 2% agarose gel.

To further investigate the contribution of NTSR1 on NET cell proliferation, adhesion, migration and invasion, we utilized NTSR1 small hairpin RNA (shRNA) to establish stable BON cell clones (N-2 and N-3) with low levels of NTSR1 expression following puromycin selection (Fig. 5A). The stable shRNA-mediated knockdown of NTSR1 decreased mRNA levels and promoter activity of interleukin-8 (IL-8), which can be induced by NTS signaling (Fig. 5A and Supplementary Fig. 4A) [32]. Treatment with SR-48692 also inhibited the promoter activity of IL-8 (Supplementary Fig. 4B). Consistent with results obtained from the above experiments using siRNA, the inhibition of cell growth and suppression of anchorage-independent growth was also noted in the cell lines expressing NTSR1 shRNA compared with control cells (Fig. 5B and 5C). In addition, an adhesion assay was performed to assess cell binding ability to the extracellular matrix which is crucial for maintaining cell viability and migration [33]. The stably-silenced NTSR1 clones demonstrated lower numbers of attached cells on type I collagen-coated plates compared with control cells (Fig. 5D and Supplementary Fig. 3B). Furthermore, BON cell clones expressing shRNA targeting NTSR1 showed significantly decreased cell migration compared to control cells (Fig. 5E). Finally, to examine the intravasation effect of NTSR1, the human Alu sequence PCR-based chick chorioallantoic membrane (CAM) assay was performed as described previously [34]. Compared to that of control, amplified bands of lower CAM inoculated with NTSR1-transfected BON cells were significantly decreased indicating that the invasive capacity of BON cells was diminished in NTSR1 knockdown clones (Fig. 5F). Collectively, these findings confirm that stable silencing of NTSR1 markedly inhibits cell proliferation, anchorage-independent growth, cell adhesion, migration and invasion suggesting an oncogenic function for NTSR1 in NET cells.

## DISCUSSION

There is emerging evidence that either NTS or NTSR1 can be utilized as a prognostic marker for various cancers due to aberrant expression noted in tumors and not detected in normal tissues, and that silencing of the genes can inhibit the tumorigenic activities in some cancer cells [15–21]. In particular, NTSR1 expression is strongly associated with a worse survival and a higher incidence of distant metastases in lung and breast cancers [17, 20]. However, the mechanisms for the expression of NTSR1 in cancer tissues including NETs, have not been well-defined. Here, we showed that NTSR1 protein is not or barely detected in 12 normal tissues but is strongly expressed in 95% of clinical NET samples (19 of 20 NETs). Similar to the above described cancers, high expression of NTSR1, noted in this study, and NTS are associated with NET progression indicating that NTSR1 may be a useful prognostic marker for NETs.

We also found that differential expression of NTSR1 and silencing of NTSR2 in NET cells is a result of promoter methylation. Previously, it was reported that DNA methylation plays an important role in the regulation of NTS expression in human cancer cells [25, 26]. Similar to the Wnt/β-catenin pathway which regulates the expression of both NTS and NTSR1 [4, 35], promoter methylation demonstrates a complicated regulatory process for NTS signaling at the level of the agonist as well as the simultaneous regulation of its receptors. In addition to hypermethylation, which is commonly found with tumor initiation and progression, hypomethylation of certain genes (e.g., urokinase plasminogen activator) is a common mechanism for the aberrant gene expression patterns in tumors, and plays a central role in human tumor progression [14, 36–38]. Similarly, the lack of NTSR1 promoter methylation is observed in all NET tissues and the promoter methylation was shown in most normal tissues in this study. These data represent that the absence of promoter methylation closely correlates with overexpression of NTSR1 in NET clinical samples. Histone modification could also be related to its transcriptional regulation similar to epigenetic repression of *DKK-1*, *DKK-3* and *WIF-1* noted in our previous study showing that silencing of these genes occurs through histone modification in NET cells, although the Wnt inhibitors are silenced by methylation of their promoters in most tumors [22]. Further work is required to elucidate the detailed mechanisms for epigenetic silencing of NTSR1 and NTSR2 in diverse tumor types.

NTS and NTSR1 are neuropeptide-receptor complexes which are frequently deregulated during tumor progression [3, 5]. In particular, NTSR1 activation induces cell proliferation, survival, migration and invasion through multiple oncogenic pathways in various cancers [3, 5]. Recently, we proposed that NTS is a mediator for NET cell growth through inhibition of NTS signaling [4]. In our current study, we confirmed the high expression of NTSR1 protein in clinical NET tissues as described above and analyzed the effect of NTSR1 on NET cell growth, adhesion, migration and invasion to better delineate the significance of NTSR1 in NET progression. In addition to cell proliferation and anchorage-independent growth, which were suppressed by NTS knockdown, NTSR1 silencing resulted in reduction of NET cell migration and invasion indicating that NTSR1 is mainly engaged in NET progression.

In summary, we have identified promoter methylation as an important molecular mechanism for the regulation of NTSR1 and NTSR2 expression. In addition, we also demonstrate high expression of NTSR1 protein in clinical NETs, and the oncogenic function of NTSR1 in NET cells. Our findings identify a potential role for NTSR1 in the growth and progression of NETs and provide a rationale for further exploration of NTSR1 as a therapeutic target for NET treatment.

## MATERIALS AND METHODS

### Immunohistochemistry

Immunostaining with NTSR1 antibody, obtained from Abcam (ab117592, Cambridge, MA), was performed as described previously [22]. The slides for paraffin-embedded tissue blocks of NETs from GI (*n* = 12), lung (*n* = 6) and thymus (*n* = 2) and normal tissues from GI (*n* = 5), lung (*n* = 5) and thymus (*n* = 2) were provided from Department of Pathology and Markey Biospecimen and Tissue Procurement Shared Resource Facility, University of Kentucky. Assessment of the stained slides was performed blindly by an experienced pathologist (EYL).

### Cell culture and treatment, siRNA transfections and lentiviral transductions

Four human NET cell lines, BON (pancreatic carcinoid), QGP-1, (pancreatic somatostatinoma), NCI-H727 (bronchial carcinoid) and UMC-11 (bronchial carcinoid) were used in this study. The cell lines were authenticated with 17 autosomal short tandem repeat loci and the sex identity locus in May 2012 at Genetica DNA Laboratories (Cincinnati, OH). BON cells were maintained in a 1:1 ratio of DMEM and F12K, supplemented with 5% FBS. QGP-1, NCI-H727 and UMC-11 cells were cultured in RPMI1640 medium with 10% FBS. Cells were grown at 37°C in a humidified 5% CO_2_ incubator. Reagents for cell treatment, a demethylating agent, 5-aza-2′-deoxycytidine and an NTSR1 antagonist, SR-48692 were purchased from Sigma (St Louis, MO) and dissolved in dimethyl sulfoxide (DMSO). Transfection with nontargeting control and SMARTPool NTSR1 siRNA (Dharmacon, Lafayette, CO) was performed using Lipofectamine RNAiMAX (Invitrogen, Carlsbad, CA) as previously described [4]. For generation of NTSR1-silenced BON cells, the shRNA lentiviruses were produced using shRNA vectors (SHGLY-NM_002531, Sigma). Cells were transduced with each virus and then selected with puromycin (2 μg/mL) as previously reported [4, 39].

### RNA isolation, reverse transcription-PCR (RT-PCR) and quantitative reverse transcription-PCR (qRT-PCR) analysis

Total RNA was isolated from the cultured NET cells using RNeasy kits according to the manufacturer’s instructions (Qiagen, Valencia, CA). Each cDNA was synthesized using High-Capacity cDNA Reverse Transcription Kit (Applied Biosystems, Foster City, CA) and total RNA for NET cells. RT-PCR analysis was performed using synthesized cDNA, HotStarTaq DNA Polymerase (Qiagen) and the primers described in Supplementary Table S1. The PCR products were analyzed on a 2% agarose gel. β-actin was used as an internal control. qRT-PCR reaction was performed using a TaqMan Gene Expression Master Mix and TaqMan probes for human NTSR1 and GAPDH according to the manufacturer’s protocol (Applied Biosystems). Expression levels were assessed by evaluating threshold cycle (Ct) values. The relative amount of mRNA expression was calculated by the comparative ΔΔCt method.

### Western blot analysis

The protein extracts for cell lysates were prepared in a Cell Lysis Buffer (Cell Signaling, Danvers, MA) containing 1 mM PMSF. Total cell lysates containing equivalent amounts of protein were separated on NuPAGE 4–12% Bis-Tris gels (Invitrogen) and transferred to PVDF membranes. The membranes were incubated with specific primary antibodies and subsequently horseradish peroxidase-conjugated secondary antibody. Following incubation with the antibody, proteins were visualized using ECL detection system (Buckinghamshire, UK). The anti-NTSR1 antibody was purchased from Santa Cruz Biotechnology (Santa Cruz, CA). The antibodies for c-Myc and Cyclin D1 were obtained from Epitomics (Burlingame, CA). The antibody for β-actin used as a loading control was from Cell Signaling.

### Methylation analysis

Methylation of 5′ regions of *NTSR1* and *NTSR2* was analyzed using MSP (methylation-specific PCR) and bisulfite sequencing analyses. Briefly, PCR was performed using bisulfite-modified genomic DNA by MethylCode Bisulfite Conversion Kit (Invitrogen) and the primers which were designed using Methyl Primer Express Software v1.0 (Applied Biosystems) and shown in Supplementary Table S1. The PCR products for MSP were visualized by 2% agarose gel. For bisulfite-sequencing, PCR products were cloned into the TOPO TA cloning vector (Invitrogen) and the plasmids from individual bacterial colonies were sequenced.

### Cell proliferation

Equal numbers of BON cells transfected with siRNA or shRNA were plated in 24-well plates. Cell proliferation was assessed at 48 h after seeding directly by cell counting using a Beckman Coulter Cell Viability Analyzer (Beckman-Coulter, Fullerton, CA).

### Soft agar assay

To measure anchorage-independent growth, BON cells were plated in growth medium containing 0.4% agarose in six-well plates onto a bottom layer of solidified 0.8% agarose. After incubation for 4 weeks, colonies were stained with crystal violet solution, washed repeatedly with distilled water and quantified using AlphaEaseFC software (Alpha Innotech Corporation, San Leandro, CA).

### Transwell migration assay

A Boyden chamber migration assay with type I collagen-coated Transwells was carried out with control and NTSR1 knockdown BON cells or BON cells treated with different concentration of SR-48692. The chambers were incubated at 37°C for 24 or 48 h, respectively, and the cells were fixed with methanol and stained with 0.5% crystal violet in 20% methanol. Activity of cell migration was quantified by counting cell numbers in four different fields under an inverted microscope.

### Luciferase reporter assays

BON cells were plated in 24-well plates and transiently transfected with the IL-8 reporter (0.4 μg) and the Renilla luciferase reporter (0.05 μg) using Lipofectamine 2000 according to the manufacturer’s instructions (Invitrogen). For SR-48692 treatment, 0 (DMSO) or 5 μM SR-48692 were treated into BON cells one day after transfection. The cells were harvested and luciferase activity was measured using a Dual-Luciferase Reporter Assay System (Promega, Madison, WI).

### Adhesion assay

A cell adhesion assay was performed to assess cell binding ability to the extracellular matrix. Briefly, 48-well plates were coated with type I collagen and washed with PBS. Equal numbers of detached BON cell clones were plated to each coated well and incubated for 15 min. After 37°C incubation, unattached cells were removed by washing with PBS, and the adherent cells were fixed, and then stained with crystal violet. The plate was washed and dried completely. Quantification was performed through counting the number of attached cells or measuring absorbance for crystal violet-stained cells at 550 nm by adding and solubilizing with 2% SDS.

### Wounding migration assay

To compare the migratory activity of stable knockdown of NTSR1, a wounding migration assay was performed with control and the two BON cell clones expressing NTSR1 shRNA. The wounded monolayers using the Culture-Insert (Ibidi, Munich, Germany) were incubated for 22 h. The cells were fixed with ice-cold methanol and stained with crystal violet. Quantification was carried out by measuring the migrated distance.

### Human Alu sequence PCR-based assay

To determine the invasive effect of NTSR1, human Alu sequence PCR-based assay was performed as described previously [34]. Stable BON cells in PBS were inoculated on artificially-generated air sacs at 1 × 10^7^ cells per chick chorioallantoic membrane (CAM) of 9 day chick embryos. After 3 d, genomic DNA was isolated from lower CAMs and then used as a template of PCR amplification. The primer sequences are shown in Supplementary Table S1. The PCR product was electrophoresed on a 2% agarose gel.

### Statistical analysis

Descriptive statistics including mean and standard deviation (SD) were calculated to summarize mRNA levels, number of cells, colony formation, migrated and attached cell number, luciferase activity and migration distance for each experiment. Bar graphs were generated to represent mean (± SD) fold changes of increase or decrease in experimental groups relative to control. Within respective experiments, comparisons between two groups were performed using two-sample *t*-tests, whereas comparisons across groups were accomplished using one-way analysis of variance models and test for linear trend of increasing dose levels or pairwise comparisons with control group were subsequently performed using contrast statements. Normality assumptions of the analysis of variance and two-sample *t*-tests for each outcome were assessed. *p*-values < 0.05 were considered statistically significant.

## SUPPLEMENTARY FIGURES AND TABLE


