# Supplementary material for: Differential expression and tumorigenic function of neurotensin receptor 1 in neuroendocrine tumor cells
Source: Oncotarget. 2015 Jul 27;6(29):26960–70. doi: 10.18632/oncotarget.4745 (PMC4694966; doi:10.18632/oncotarget.4745)
Supplement: Supplementary file 1 [file oncotarget-06-26960-s001.pdf]

SUPPLEMENTARY FIGURES AND TABLE

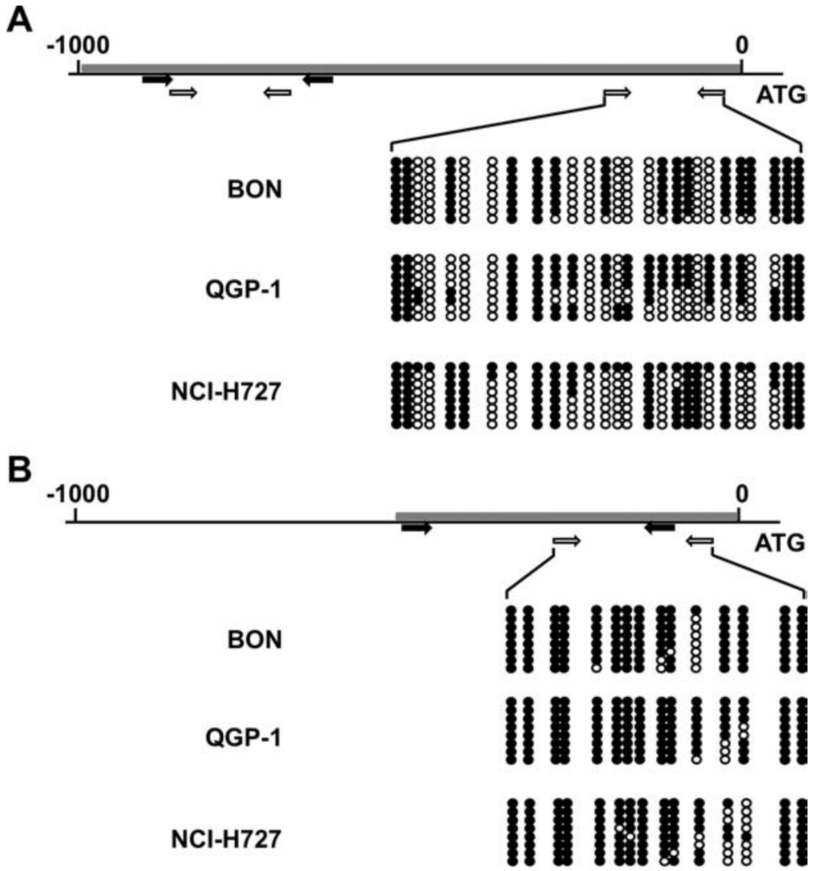

Supplementary Figure S1: Sequencing analysis of the *NTSR1* and *NTSR2* MSP products of NET cells. A. Sequencing analysis of *NTSR1* promoters by MSP using NTSR1B primer set in BON, QGP-1 and NCI-H727 cells. Each row of circles represents the DNA sequence of an individual clone; closed and open circles indicate methylated and unmethylated CpG sites, respectively. Bold gray lines are candidate CpG island regions searched by Methyl Primer Express Software v1.0 (Applied Biosystems). The open, and closed arrows below the CpG islands represent the primers for MSP and bisulfite sequencing, respectively. **B.** MSP sequencing analysis of the *NTSR2* CpG islands in the NET cells as described above.

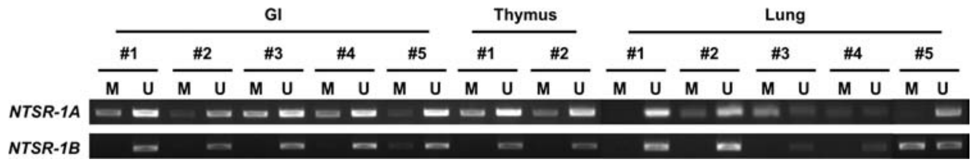

Supplementary Figure S2: MSP analysis of the *NTSR1* gene promoter region in normal tissues. The PCR products using respective two NTSR1 primer pairs (NTSR1A and NTSR1B) specific for the methylated (M) and unmethylated (U) DNA were visualized by 2% agarose gel. The normal tissues including GI, thymus and lung tissues for genomic DNA were provided from Markey Biospecimen and Tissue Procurement Shared Resource Facility, University of Kentucky.

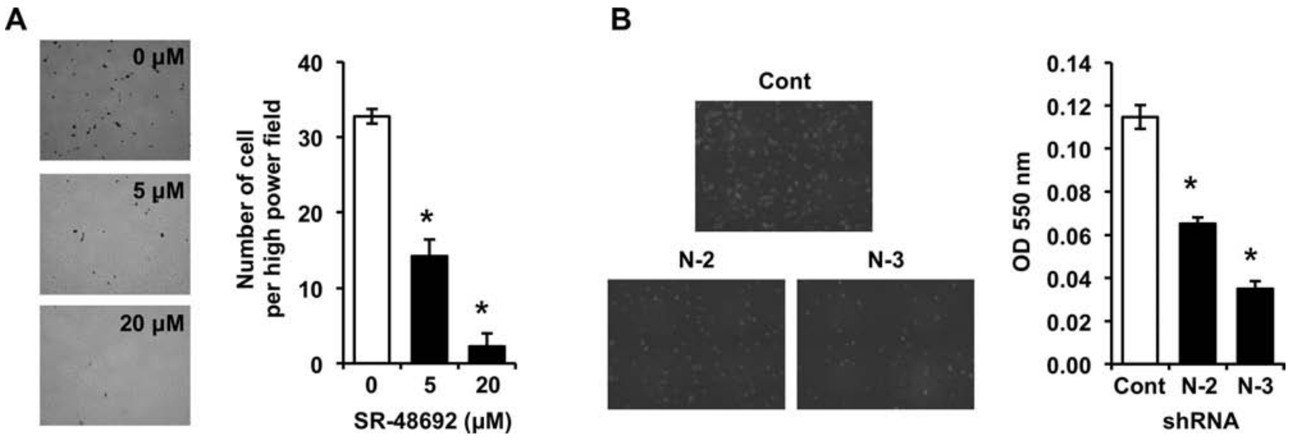

Supplementary Figure S3: Effects of pharmacologic inhibition and stable silencing for NTSR1 on NET cell migration and attachment, respectively. **A.** Transwell migration assays were performed using BON cells treated with different concentrations of SR-48692, an NTSR1 antagonist over 24 h. Microscopic examination of the migrated BON cells (left); the number of migrated cells was counted in four different fields with an inverted microscope (right; *$p < 0.05$ vs. DMSO). **B.** Microscopic examination of the attached BON cell clones (N-2 and N-3) in 48 well plates coated with collagen I for 15 min (left). The absorbance for crystal violet-stained cells was measured at 550 nm (right; *$p < 0.05$ vs. control shRNA).

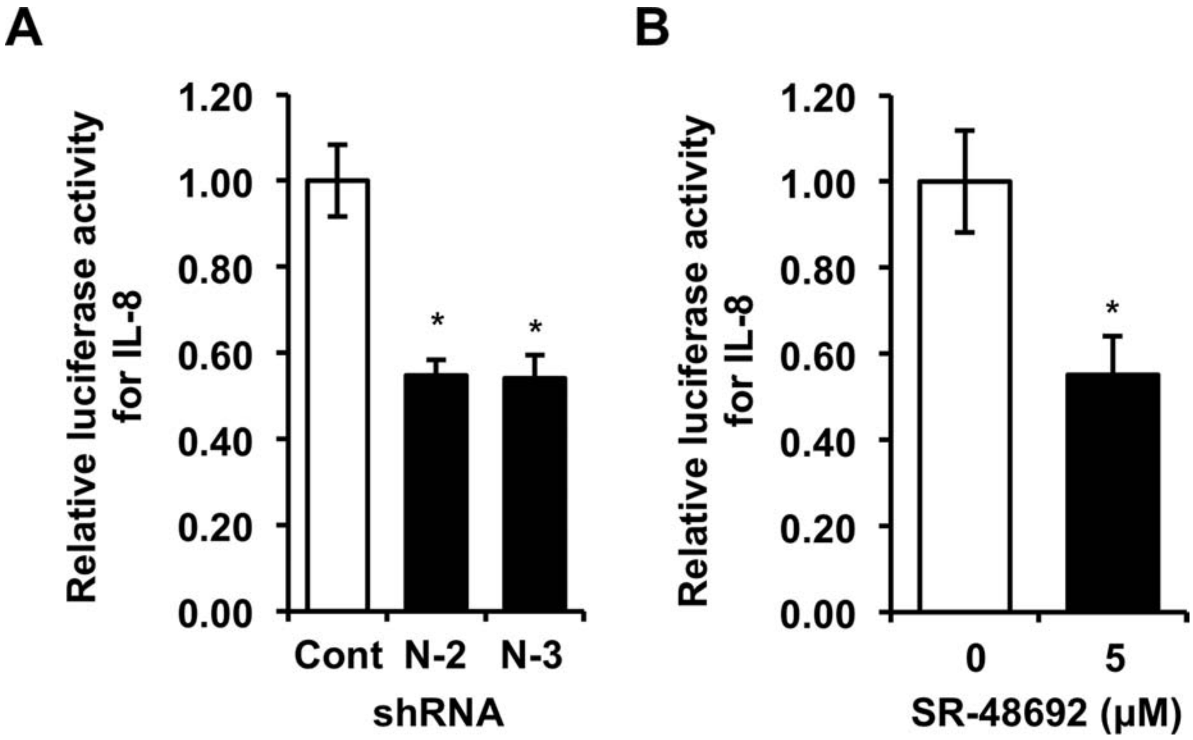

Supplementary Figure S4: Effects of stable knockdown or pharmacologic blockade of NTSR1 on IL-8 promoter activities in BON cells. A. Relative luciferase activity of an IL-8 promoter reporter in NTSR1 knockdown BON cell clones. BON cell clones were plated in 24-well plates and transiently transfected with an IL-8 promoter reporter and a Renilla luciferase reporter. Luciferase activity was measured 2 d after transfection (*$p < 0.05$ vs. control shRNA). **B.** Relative IL-8 promoter activity of BON cells treated with 0 (DMSO) or 5 μM SR-48692 (*$p < 0.05$ vs. DMSO).

Supplementary Table S1. Primer sequences for PCR reaction (F, Forward; R, Reverse; MSP, Methylation-Specific PCR; M, Methylated DNA; U, Unmethylated DNA; BS, Bisulfite-Sequencing)

Target	Experiment	Primer Name	Primer sequences
NTSR1	RT-PCR	NTSR1 F	TCATCGCCTTTGTGGTCTGCT
		NTSR1 R	TGGTTGCTGGACACGCTGTCG
NTSR2	RT-PCR	NTSR2 F	GTCTCCTCAGCTTCATCGTAT
		NTSR2 R	TCCCCAAAGCCTGAAGCTGTA
NTSR3	RT-PCR	NTSR3 F	AGAATGGTCGAGACTATGTTG
		NTSR3 R	AAGAGCTATTCCAAGAGGTCC
NTS	RT-PCR	NTS F	GATGATGGCAGGAATGAAAATCCAG
		NTS R	GTTGAAAAGCCCTGCTGTGACAGA
β-actin	RT-PCR	β-actin F	TCACCAACTGGGACGACATG
		β-actin R	ACCGGAGTCCATCACGATG
NTSR1	MSP M	MSP NTSR1A M F	GGTCGGTTTAATTAGTTGC
		MSP NTSR1A M R	CGAATATCGTACACCAAAC
NTSR1	MSP U	MSP NTSR1A U F	GGGGTTGGTTTAATTAGTTGT
		MSP NTSR1A U R	ACAAATATCATACACCAAACC
NTSR1	MSP M	MSP NTSR1B M F	TTGGAATTCGTGGTAAGC
		MSP NTSR1B M R	GTCTCAAACGAAAACCGATA
NTSR1	MSP U	MSP NTSR1B U F	TATTTGGAATTTGTGGTAAGT
		MSP NTSR1B U R	ATCTCAAACAAAAACCAATAAAC
NTSR2	MSP M	MSP NTSR2 M F	GTGGAGTTCGGTTTAATTC
		MSP NTSR2 M R	ACTACCCGAAATCTAAACG
NTSR2	MSP U	MSP NTSR2 U F	GGTGGAGTTTGGTTTAATTT
		MSP NTSR2 U R	CACTACCCAAAATCTAAACA
NTSR1	BS	BS NTSR1 F	TTGTGGATATTTAGGAGTGGG
		BS NTSR1 R	CTCCAAAAAACCAAAATTCC
NTSR2	BS	BS NTSR2 F	TGTTGGGAAAGTTTTTTTTAAG
		BS NTSR2 R	AAACACCTCCTCTTCTCTAAAAA
c-Myc	RT-PCR	c-Myc F	TGAAAGGCTCTCCTTGCAGC
		c-Myc R	GCTGGTAGAAGTTCTCCTCC
CyclinD1	RT-PCR	CyclinD1 F	ATGTGTGCAGAAGGAGGTCC
		CyclinD1 R	CTTAGAGGCCACGAACATGC
IL-8	RT-PCR	IL-8 F	CATGACTTCCAAGCTGGCCG
		IL-8 R	AATTTTTTTATGAATTCTCAGCCCTC
Alu	PCR	Alu F	ACGCCTGTAATCCCAGCACTT
		Alu R	TCGCCCAGGCTGGAGTGCA
